# Real-World Symptom Trajectories in Adolescents With and Without Suicide Risk Receiving Care from Rula Health: Retrospective Study

**DOI:** 10.2196/81444

**Published:** 2025-10-15

**Authors:** Lara Baez, Kelsey L McAlister, Douglas Newton, Sam Seiniger, Allie Woodhouse, Jennifer Huberty

**Affiliations:** 1 Fit Minded Inc Phoenix, AZ United States; 2 Rula Health Santa Clara, CA United States

**Keywords:** digital therapy, telehealth, measurement-based care, suicidality, teenagers, youth

## Abstract

**Background:**

More than 5 million US adolescents experience mental or behavioral health conditions, yet two-thirds remain untreated, and suicide is the second leading cause of death. These gaps highlight the urgent need for accessible care. Digital mental health interventions that integrate measurement-based care (MBC) and personalized mental health care provider matching offer a promising solution, but few studies have examined their real-world impact among adolescents at elevated suicide risk.

**Objective:**

This study aims to evaluate symptom improvements among adolescents with and without elevated suicide risk receiving care from Rula Health, an MBC-based digital mental health intervention with personalized intake through mental health care provider matching. We aimed to (1) compare baseline demographic and clinical characteristics between adolescents with and without elevated suicide risk at intake and (2) examine depression and anxiety symptom trajectories over the first 12 visits between adolescents with and without elevated suicide risk at intake.

**Methods:**

We conducted a retrospective analysis of real-world clinical data from adolescents who received mental health services through Rula Health. Adolescents were classified as no suicide risk or elevated suicide risk based on the Columbia-Suicide Severity Rating Scale. Depression and anxiety symptoms were assessed using the Patient Health Questionnaire-9 (PHQ-9) and the Generalized Anxiety Disorder-7 (GAD-7) at baseline and before each session. Minimal clinically important differences were defined as a reduction of 5 more points for PHQ-9 and 4 or more points for GAD-7. Symptom changes were examined up to 12 visits. We used *t* tests and chi-square tests to compare baseline characteristics between suicide risk groups and linear mixed-effects models (adjusted for demographics and clinical factors) to assess symptom change and trajectory differences over time.

**Results:**

The sample included 3533 adolescents in the no suicide risk group and 2712 in the elevated suicide risk group. The elevated suicide risk group had a greater proportion of female adolescents, younger adolescents (*P*<.001), non-Hispanic individuals (*P*=.002), and those with a primary depressive diagnosis, comorbid conditions, psychiatric involvement, and higher baseline PHQ-9 and GAD-7 scores (*P*<.001). The no suicide risk group attended more sessions and stayed in care longer (*P*<.001). Depression and anxiety scores decreased over visits (PHQ-9: B=–0.39; *P*<.001; GAD-7: B=–0.35; *P*<.001), with average improvements exceeding minimal clinically important difference thresholds. The elevated suicide risk group’s depression and anxiety symptoms decreased at a higher rate than those of the no suicide risk group (PHQ-9: B=–0.32; *P*<.001; GAD-7: B=–0.18; *P*<.001).

**Conclusions:**

Adolescents with elevated suicide risk showed greater and faster improvement in depression and anxiety symptoms, reaching similar levels as those without elevated suicide risk by 12 visits. Rula Health’s model can support high-risk youth in real-world settings. Future research should assess the impact of MBC and mental health care provider matching, including study designs that isolate their specific effects on outcomes.

## Introduction

### Background

Adolescence is a critical developmental period marked by heightened vulnerability to mental health disorders, with more than 5 million adolescents in the United States diagnosed with a mental or behavioral condition, most commonly depression and anxiety [1]. Depression and anxiety symptoms can deeply impact how adolescents function in their daily lives, affecting their relationships, academic performance, and overall well-being [2,3]. Even more concerning is the fact that these symptoms are strongly linked to suicide, the second leading cause of death among adolescents, with rates rising to 62% from 2007 to 2021, even as they plateau or decline in other populations in the United States [4-6]. One in 5 high school students has seriously considered suicide, with more than 1 in 10 reporting that they attempted suicide [7]. Rates of hospitalization for suicide-related behaviors in adolescents have sharply increased, placing added strain on families, school systems, and the broader health care system [8,9]. Despite the urgency of these trends, 61% of adolescents with mental health needs report difficulty accessing treatment due to barriers, such as mental health care provider shortages, long waitlists, stigma, and cost [1]. These realities underscore the urgent need for timely, accessible, and effective mental health interventions.

Digital mental health interventions (DMHIs), including virtual therapy and psychiatric services, have emerged as a scalable and flexible solution to address the growing demand for adolescent mental health care [10]. By offering therapy and psychiatric services through virtual platforms, DMHIs can help overcome many of the barriers that limit access to traditional in-person care, such as geographic limitations, mental health care provider shortages, long wait times, and stigma [11], though access may remain uneven due to disparities in broadband connectivity, device availability, and digital literacy [12,13]. These platforms are particularly well-suited to adolescents, who are often comfortable with technology and may prefer the privacy and convenience of engaging in care from home [14,15]. In addition to expanding access, DMHIs offer the potential to deliver more personalized care by adapting treatment to individual needs, goals, and preferences in a flexible, user-centered format. While emerging evidence suggests that DMHIs can reduce depression and anxiety symptoms among youth experiencing suicidal thoughts [16,17], their effectiveness depends on how care is tailored and monitored over time. Consequently, there is a growing need for digital platforms to deliver care that is both personalized and informed by structured symptom monitoring.

Measurement-based care (MBC) is an evidence-based approach that uses standardized assessments to monitor symptoms and guide clinical decision-making throughout treatment [18]. It has been linked to improved outcomes and stronger therapeutic alliance across diverse mental health settings [18,19]. Although some studies in routine outpatient care have found modest or variable effects [20], and meta-analytic evidence suggests MBC does not consistently improve symptom outcomes [21], this variability highlights the importance of examining MBC in populations where its impact may be most critical. For adolescents at elevated suicide risk, MBC may offer a mechanism to detect rapid symptom changes, support timely adjustments in treatment, and complement clinical decision-making in high-stakes contexts. In parallel, personalization strategies, such as offering a curated list of mental health care provider options tailored to clinical needs and individual preferences, may help ensure that care is well-aligned from the outset [22,23]. DMHIs are well-positioned to implement both approaches efficiently, using technology to support structured symptom tracking and individualized mental health care provider selection at scale.

Despite the promise of DMHIs, including virtually delivered therapy and psychiatry, important gaps remain, particularly for adolescents at elevated risk for suicide. DMHIs have demonstrated effectiveness in reducing depression and anxiety symptoms among adolescents in both clinical trials and real-world settings [24-26]. However, relatively few studies have assessed whether adolescents with elevated suicide risk experience similar patterns of symptom improvement compared to those without such risk [27]. While emerging research on DMHIs has begun to include adolescents with elevated risk for suicide [16,28], most studies do not stratify outcomes by suicide risk status, limiting our understanding of whether this subgroup benefits equally from care. Moreover, a recent systematic review and multiple studies show that adolescents with suicidal thoughts or behaviors are often excluded from DMHI studies for safety reasons, which limits generalizability and contributes to the lack of real-world evidence for this high-risk group [16,29-31]. In addition, little is known about how digital care models that incorporate both MBC and intake personalization perform for adolescents at elevated suicide risk. Addressing these gaps is essential for designing digital mental health services that are not only scalable but also safe, effective, and equitable for the youth who face the greatest clinical challenges.

### Objectives

The purpose of this study was to evaluate symptom improvements among adolescents with and without elevated suicide risk receiving care from Rula Health, an MBC-based DMHI with personalized intake through mental health care provider matching. We aimed to (1) compare baseline demographic and clinical characteristics between adolescents with and without elevated suicide risk at intake and (2) examine depression and anxiety symptom trajectories over the first 12 visits between adolescents with and without elevated suicide risk at intake.

## Methods

### Study Design and Participants

This retrospective study examined secondary data from Rula Health, a DMHI that connects individuals across the United States with licensed therapists and psychiatry providers. Rula Health’s care model is grounded in MBC and personalized treatment approaches aimed at improving clinical outcomes. Adolescents (aged 12-17 years) were included in this study if they received therapy and psychiatry services from Rula Health between August 24, 2022, and June 30, 2025, and completed the Columbia-Suicide Severity Rating Scale (C-SSRS) during their initial visit as part of standard clinical procedures. Patients were sent the C-SSRS when their baseline total Patient Health Questionnaire-9 (PHQ-9; refer to the description mentioned subsequently in the Study Measures section) score was above 15 or if PHQ-9 item 9 indicated risk. In this study, participants were classified as no suicide risk (total C-SSRS score=0) or elevated suicide risk (total C-SSRS score ≥1) [32]. This threshold aligned with clinical practice, where any endorsement of suicidal ideation or behavior on the C-SSRS was considered a positive screen requiring further evaluation [32,33].

### Ethical Considerations

Before beginning care, all individuals receiving services through Rula Health were required to provide informed consent. The consent process included an explanation of the nature and scope of the services, confidentiality protections, and how personal data may be used. In addition to the informed consent process, patients reviewed and accepted Rula Health’s privacy policy, which detailed how personal information was collected, used, and protected in accordance with applicable regulatory standards. In accordance with US regulations governing research on human participants, this study was determined to be exempt from further informed consent requirements. Therefore, no additional consent procedures were conducted. The protocol was reviewed and approved by the institutional review board of the Biomedical Research Alliance of New York (25-035-2061). No compensation was provided to individuals whose data were included in this study.

### Treatment

Rula Health’s therapy and psychiatry care models have been detailed elsewhere [34]. Patients accessed Rula Health through a range of referral sources, including primary care providers, insurance partners, employer-sponsored mental health benefits, health care organizations, and direct online searches. The platform is designed to simplify access to mental health services while supporting patient autonomy in mental health care provider selection. After completing a brief intake questionnaire, individuals were matched with a curated list of licensed therapists or psychiatric providers based on their clinical needs, insurance coverage, and personal preferences (eg, provider specialty, background, and availability), from which they selected the mental health care provider who best fits their needs.

Treatment was delivered virtually via secure video sessions. For therapy, initial visits focused on collaboratively developing a personalized treatment plan that includes therapy goals, intervention strategies, and a schedule of care. Therapists drew from a range of evidence-based modalities, such as cognitive behavioral therapy, dialectical behavior therapy, and acceptance and commitment therapy, tailoring care to each patient’s needs. Data related to fidelity or adherence to specific evidence-based treatment protocols were not collected. For psychiatric care, initial visits included a comprehensive review of medical and psychiatric history, discussion of presenting concerns, and the development of an individualized treatment plan that may include medication management. Provider quality was monitored through clinical tools and quality assurance protocols. For example, mental health care provider quality was monitored by clinical quality specialists, who were licensed clinicians who provided consultation and feedback to providers. Providers were also flagged for issues related to documentation quality and were given targeted feedback and ongoing monitoring until issues were resolved.

As part of MBC, patients completed standardized symptom assessments at regular intervals, and mental health care providers were encouraged to use these data to inform treatment decisions. For therapy, these scores supported progress tracking and treatment adjustment, while in psychiatry, they informed decisions related to medication, referrals, or other interventions. Rula Health implemented rigorous safety protocols for managing elevated suicide risk, including proactive risk identification (eg, using the C-SSRS), crisis response, and care coordination. The patient safety and clinical risk (PSCR) team guided providers to ensure best practices in clinical risk management and patient safety. When elevated risk was detected, mental health care providers developed and documented a safety plan and reached out to the PSCR team for consultation and help with referrals. Patients also had access to a 24×7 crisis support line. When a patient accessed the crisis line, the PSCR team followed up with the mental health care provider and helped initiate referrals when appropriate.

### Study Measures

#### Overview

At the start of care, patients provided demographic information, such as age, sex assigned at birth, race, and ethnicity. Primary diagnoses were documented by the treating provider. Beginning August 20, 2020, measures of mental health symptoms were administered before each scheduled visit or up to once weekly. As of December 8, 2024, the frequency of assessment shifted to biweekly. This analysis focused on symptom data related to depression and anxiety.

#### Depression

Depression symptoms over the past 2 weeks were assessed using the first 9 items of the PHQ-9 modified for adolescents [35,36]. Patients rated each item on a 4-point Likert scale ranging from 0 (*not at all*) to 3 (*nearly every day*), yielding a total score from 0 to 27. Higher scores indicated greater levels of depression. The PHQ-9 has demonstrated strong internal consistency (Cronbach α=0.89) and excellent test-retest reliability (Pearson r=0.84) [37,38]. Results were contextualized using minimal clinically important difference (MCID) in the PHQ-9, the change in symptom severity that is perceived as meaningful by patients (defined as a reduction of ≥5 points [39]).

#### Anxiety

Anxiety symptoms were assessed using the Generalized Anxiety Disorder-7 (GAD-7), a 7-item tool that assesses symptom severity over the past 2 weeks [40]. Each item is rated on a 4-point Likert scale from 0 (*not at all*) to 3 (*nearly every day*), yielding a total score from 0 to 21. Higher scores indicate greater levels of anxiety. The GAD-7 demonstrates high internal consistency (Cronbach α=0.89-0.92) and strong test-retest reliability (intraclass correlation=0.83) [40-42]. Results were also contextualized using MCID in the GAD-7, defined as a reduction of 4 or more points [43].

### Statistical Analyses

Data were limited to the first 12 visits because this is a common length of treatment for adolescent mood and anxiety disorders [44] and represents a relative episode of care at Rula Health. Demographic and baseline descriptive statistics were calculated for those in the no suicide risk (ie, total C-SSRS score=0) and those in the elevated suicide risk group (ie, total C-SSRS score ≥1), and differences between the groups were tested using independent sample 2-tailed *t* tests for continuous variables and chi-square tests for categorical variables. Effect sizes were calculated using Cohen d for the *t* tests and Cramér V for the chi-square test.

To examine changes in depression and anxiety symptoms over time and determine whether trajectories differed by suicide risk group, linear mixed-effects models were fit. Separate models were fitted for depression and anxiety outcomes. The following elements were added subsequently and tested using the likelihood ratio test to determine the best-fitting model: main effect of visit number, main effect of baseline suicide risk group, and the visit number×suicide risk group interaction (this tested whether the rate of change in symptoms differed between groups). All models were adjusted for covariates, including patient age, sex, race, ethnicity, primary diagnosis, and comorbid diagnosis (ie, whether patients had at least 2 diagnoses or not). A random intercept was included in all models to account for repeated measures nested within patients. Random slopes for visit number were also included and retained if they improved model fit based on the likelihood ratio test. If random slopes models did not converge, models were refitted assuming an independent covariance structure (ie, uncorrelated random effects). Significance of fixed effects was assessed using the Satterthwaite approximation of degrees of freedom [45]. To visualize model-predicted symptom trajectories, model-predicted depression and anxiety scores over visits were calculated using the fixed effects from each best-fitting model. Predicted values were calculated for each suicide risk group at each visit, and 95% CIs were derived from the model-based SEs of the predicted means. Assumptions, including multicollinearity and normality of residuals, were checked (Multimedia Appendix 1 [46]). All analyses were conducted in R (version 4.3.1; R Foundation for Statistical Computing; [47]).

## Results

### Descriptive Statistics and Group Comparisons

Both the no suicide risk and elevated suicide risk groups consisted of individuals, on average, aged 15 years (SD 1.46), and they were mostly female, White, and non-Hispanic. The no suicide risk and elevated suicide risk groups differed on several demographic and clinical characteristics. The elevated suicide risk group had a greater proportion of female adolescents, younger adolescents (aged 12-14 years), non-Hispanic adolescents, and adolescents with a primary diagnosis of a depressive disorder. These differences were largest in age (Cramér V=0.17) and primary diagnosis (Cramér V=0.17). The elevated suicide risk group also had a greater proportion of adolescents with a comorbid diagnosis and adolescents receiving psychiatry services and also greater baseline PHQ-9 and GAD-7 scores. These differences were largest for baseline PHQ-9 score (Cohen *d*=–0.90) and baseline GAD-7 score (Cohen *d*=–0.61). The no suicide risk group had a greater number of visits up to 12 and were in treatment for a longer time on average (Table 1).

**Table 1:** Descriptive statistics of demographic, baseline, and treatment characteristics in the no suicide risk and elevated suicide risk groups^a^.

Demographic characteristic		No suicide risk (n=3533)	Elevated suicide risk (n=2712)	Chi-square (*df*)		*t* test (*df*)		Effect size		*P* value	
**Sex, n/N (%)**					45.1 (1)		—^b^		0.086^c^		<.001
	Female	2247/3507 (64.07)	1918/2657 (72.19)								
	Male	1260/3507 (35.93)	739/2657 (27.81)								
**Race, n/N (%)**					2.2 (5)		—		0.026^c^		.82
	African American or Black	173/1739 (9.95)	141/1432 (9.85)								
	American Indian or Alaska Native	17/1739 (0.98)	17/1432 (1.19)								
	Asian	134/1739 (7.71)	129/1432 (9.01)								
	Mixed race	415/1739 (23.88)	341/1432 (23.81)								
	Native Hawaiian or Pacific Islander	39/1,739 (2.24)	33 (2.30)								
	White	960/1739 (55.24)	771/1432 (53.84)								
Ethnicity—Hispanic, n/N (%)		850/2014 (42.20)	591/1591 (37.15)	9.3 (1)		—		0.051^c^		.002	
**Age (y; categorical), n/N (%)**					170.7 (5)		—		0.17^c^		<.001
	12	191/3533 (5.41)	275/2712 (10.1)								
	13	245/3533 (6.93)	326/2712 (12.02)								
	14	281/3533 (7.95)	341/2712 (12.57)								
	15	597/3533 (16.90)	425/2712 (15.67)								
	16	1045/3533 (29.58)	633/2712 (23.34)								
	17	1174/3533 (33.23)	712/2712 (26.25)								
Age (y, continuous), mean (SD)		15.58 (1.46)	15.09/2712 (1.67)	—		12.17 (5395.3)		0.32^d^		<.001	
**Primary diagnosis, n/N (%)**					186.6 (3)		—		0.17^c^		<.001
	Depressive disorders	665/3456 (19.24)	916/2660 (34.44)								
	Anxiety disorder	1121/3456 (32.44)	643/2660 (24.17)								
	Trauma- and stress-related disorders	928/3456 (26.85)	592/2660 (22.26)								
	Other	742/3456 (21.47)	509/2660 (19.14)								
Comorbid diagnosis, n/N (%)		1094/3534 (30.97)	1071/2713 (39.49)	48.9 (1)		—		0.089^c^		<.001	
Psychiatry patient, n/N (%)		113/3531 (3.20)	179/2712 (6.60)	39.1 (1)		—		0.080^c^		<.001	
Baseline Patient Health Questionnaire-9 score, mean (SD)		9.35 (6.95)	15.11 (5.64)	—		–16.15 (6223.4)		–0.90^d^		<.001	
Baseline Generalized Anxiety Disorder-7 score, mean (SD)		8.60 (5.71)	11.83 (4.79)	—		–24.26 (6191)		–0.61^d^		<.001	
Total visits up to 12, mean (SD)		7.32 (4.32)	6.54 (4.21)	—		7.23 (5900.6)		0.18^d^		<.001	
Length of treatment (wk), mean (SD)		12.03 (11.79)	9.70 (9.92)	—		8.50 (6190)		0.21^d^		<.001	

^a^Mean (SD) was reported for continuous variables, and percentages were reported for categorical variables. *t* tests were used to compare continuous variables, and chi-square tests were used for categorical variables.

^b^Not applicable.

^c^Refers to Cramér V values.

^d^Refers to Cohen *d* values.

### Trajectories of Depression and Anxiety Symptoms

**Figure 1:**
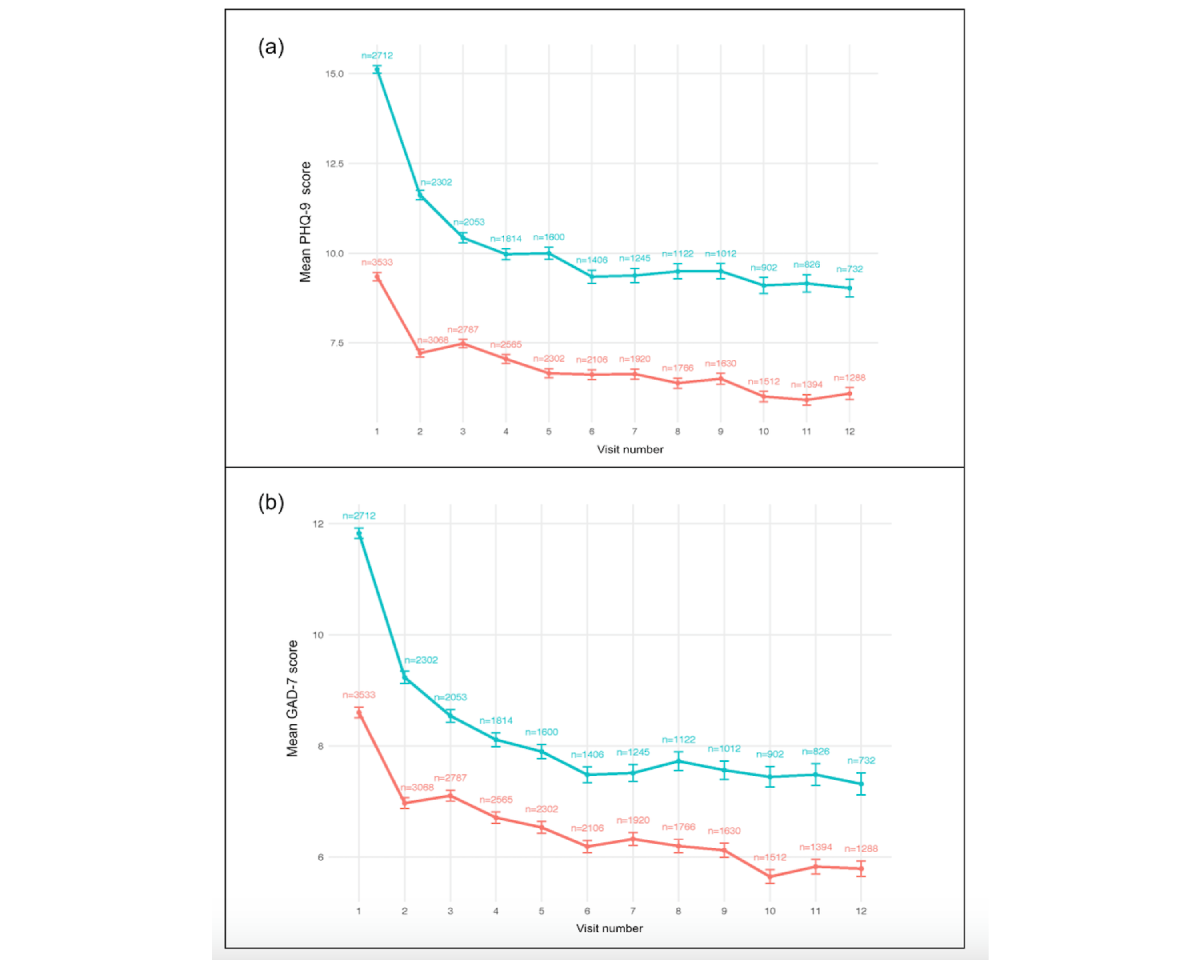
Raw trajectories of depression (A) and anxiety (B) symptoms by baseline suicide risk group.

Raw trajectories of PHQ-9 and GAD-7 for both suicide risk groups are presented in Figure 1. In Figure 1, the blue line represents adolescents with elevated suicide risk and the red line represents those with no suicide risk; “n” indicates the number of patient observations at each visit. Successively including a main effect of visit number, a main effect of suicide risk group, and an interaction between visit number and suicide risk group improved model fit at each step (Table S1 in Multimedia Appendix 1). At all steps, a random intercept-only model was compared to a model that included a random slope. At all steps, models with random slopes fit best, as indicated by the likelihood ratio test. Thus, the best-fitting models for both PHQ-9 and GAD-7 trajectories were the final models that included random slopes for visit number and an interaction of suicide risk group and visit number.

**Figure 2:**
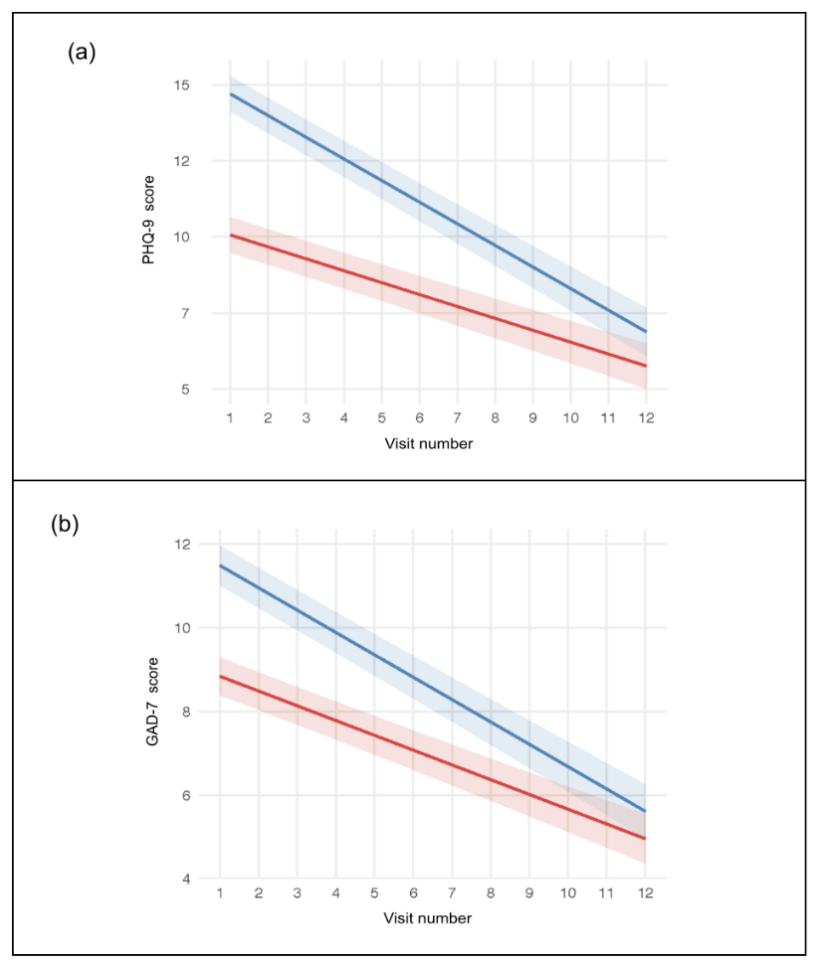
Depression (A) and anxiety (B) model-estimated symptom trajectories by suicide risk group.

Results of the best-fitting models adjusted for covariates showed that, on average, adolescents’ depression and anxiety scores decreased over visits (PHQ-9: B=–0.39; *P*<.001; GAD-7: B=–0.35; *P*<.001) and that, on average, the elevated suicide risk group had greater depression and anxiety scores over time than the no suicide risk group (PHQ-9: B=4.95; *P*<.001; GAD-7: B=2.83; *P*<.001). The significant visit number×suicide risk group interaction terms showed that the elevated suicide risk group’s depression and anxiety symptoms decreased at a higher rate than the no suicide risk group (PHQ-9: B=–0.32; *P*<.001; GAD-7: B=–0.18; *P*<.001; Table 2; Figure 2). As shown in Figure 2, model-estimated trajectories were adjusted for age, gender, race, ethnicity, primary diagnosis, and comorbidity; the blue line represents the elevated suicide risk group and the red line represents the no suicide risk group, with shaded areas denoting 95% CIs. The covariate effects of these models are presented in Table S2 in Multimedia Appendix 1. Briefly, older age and comorbidities were associated with higher symptom scores, while male sex and African American or Black race (vs White race) were associated with lower scores. Depressive disorders predicted higher depression scores, and anxiety disorders predicted higher anxiety scores.

To contextualize our findings, the no suicide risk group’s slope of –0.39 on the PHQ-9 corresponded to an average reduction in PHQ-9 of 4.68 points over 12 visits, and the elevated suicide risk group’s slope of –0.39 to –0.36 (=–0.75) corresponded to an average reduction in PHQ-9 of 9 points over 12 visits. The no suicide risk group’s slope of –0.35 on the GAD-7 corresponded to an average reduction in GAD-7 score of 4.2 points over 12 visits, and the elevated suicide risk group’s slope of –0.35 to –0.19 (=–0.54) corresponded to an average reduction in GAD-7 of 6.48 points over 12 visits. Average reductions in the no suicide risk group approached the MCID threshold for PHQ-9 (at least a 5-point reduction) and exceeded the threshold for GAD-7 (at least a 4-point reduction). Average reductions in the elevated suicide risk group were well over these established MCID thresholds.

**Table 2:** Results (fixed effects) for best-fitting models for depression and anxiety symptom trajectories.

Outcome		Unadjusted model			Adjusted model^a^					
		B (SE)	*t* test (*df*)	*P* value		B (SE)	*t* test (*df*)		*P* value	
**Patient Health Questionnaire-9 score**										
	Intercept	9.11 (0.10)	87.81 (7882.74)	<.001	10.36 (0.30)			34.75 (4039.90)		<.001
	Visit number	–0.39 (0.018)	–21.64 (2162.71)	<.001	–0.39 (0.024)			–16.37 (1171.80)		<.001
	Suicide risk group (at risk)	5.55 (0.15)	35.21 (7954.48)	<.001	4.95 (0.22)			22.88 (4015.3)		<.001
	Visit number×suicide risk group (at risk)	–0.36 (0.027)	–13.18 (2278.87)	<.001	–0.32 (0.036)			–8.76 (1221.36)		<.001
**Generalized Anxiety Disorder-7 score**										
	Intercept	8.45 (0.085)	99.45 (8093.66)	<.001	9.12 (0.23)			39.48 (3721.34)		<.001
	Visit number	–0.35 (0.015)	–23.61 (2316.27)	<.001	–0.35 (0.020)			–17.87 (1270.67)		<.001
	Suicide risk group (at risk)	3.05 (0.13)	23.59 (8165.40)	<.001	2.83 (0.18)			15.74 (4175.83)		<.001
	Visit number×suicide risk group (at risk)	–0.19 (0.022)	–8.55 (2443.60)	<.001	–0.18 (0.030)			–6.03 (1325. 80)		<.001

^a^Adjusted for age, sex, race, ethnicity, primary diagnosis, and comorbidity.

## Discussion

### Principal Findings

The purpose of this study was to evaluate symptom improvements among adolescents with and without elevated suicide risk receiving care from Rula Health, an MBC-based DMHI with personalized intake through mental health care provider matching. We aimed to (1) compare baseline demographic and clinical characteristics between adolescents with and without elevated suicide risk at intake and (2) examine depression and anxiety symptom trajectories over the first 12 visits between adolescents with and without elevated suicide risk at intake. The elevated suicide risk group had more female adolescents, younger adolescents, non-Hispanic adolescents, and adolescents with depressive disorder diagnoses. The elevated suicide risk group had more adolescents with comorbid diagnoses, more adolescents receiving psychiatry services, and greater baseline depression and anxiety scores. The no suicide risk group attended more visits (up to 12) and was in treatment for a longer time than the elevated suicide risk group. Across all adolescents, depression and anxiety scores decreased over 12 visits. The elevated suicide risk group’s depression and anxiety symptoms decreased at a higher rate than the no suicide risk group, and the elevated suicide risk group achieved similar depression and anxiety scores as the no risk group by 12 visits.

Among adolescents who received care through Rula Health, those with elevated suicide risk at intake differed from their no-risk peers. This group included higher proportions of female adolescents, non-Hispanic adolescents, and younger adolescents. These differences largely mirror national epidemiological patterns. For example, data from US adolescents, including national-level surveys, consistently show that adolescent girls report higher rates of suicidal ideation and attempts than boys [48-51]. National survey data also show that non-Hispanic White adolescents are more likely to report suicidal ideation than Hispanic youth [51,52], which aligns with our finding that elevated risk was more common among non-Hispanic participants. The overrepresentation of non-Hispanic adolescents aligns with research indicating that Hispanic and Latino identity may be protective against suicidality, possibly due to cultural values, such as familism and religiosity [53,54], and that Hispanic and Latino youth face barriers to accessing mental health care [55,56]. Interestingly, greater proportions of younger adolescents were in the elevated suicide risk group. This is different from national surveillance data and previous longitudinal studies showing that risk for suicidal ideation, suicide attempts, and death by suicide increases from early to midadolescence [57,58]. Emerging evidence points to a sharp rise in suicidality among preteenagers, which may help explain increased help seeking in this group [59,60]. The presence of high-risk youth across multiple demographic groups in Rula Health’s patient population is encouraging, suggesting that this type of DMHI may help expand access to care for adolescents at greatest risk. Building on this, future research should examine how Rula Health’s personalized intake and mental health care provider matching systems influence access and outcomes across sex, ethnicity, and age groups.

Adolescents with elevated suicide risk at intake presented with greater rates of depressive disorder as well as greater clinical complexity as indicated by higher rates of comorbid diagnoses, greater use of psychiatric services, and more severe baseline depression and anxiety symptoms. Major depressive disorder and co-occurring conditions are strongly linked to heightened suicide risk [61,62]. Clinically complex adolescents at risk of suicide need more intensive, frequent, and specialized care [63,64]. However, more than two-thirds of adolescents who die by suicide had no treatment contact within the month before death, highlighting the critical gaps in traditional service access and engagement [65]. Our findings point to the critical importance of DMHIs, such as Rula Health, in reaching and serving clinically complex adolescents who may otherwise face significant barriers to accessing timely evidence-based care [66,67]. Future research should examine whether there is an optimal type of treatment or therapy modality for adolescents with elevated suicide risk, high symptom severity, and multiple comorbidities.

The individuals in the elevated suicide risk group attended fewer sessions and were in treatment for less time than the individuals in the no suicide risk group. Adolescents at risk of suicide face attitudinal barriers to care, such as shame and stigma, which can impact how they engage with mental health services, including contributing to premature dropout [68-70]. Alternatively, some adolescents in the elevated suicide risk group may have had fewer sessions because they were transitioned appropriately to higher levels of care or experienced a more rapid reduction in symptoms, warranting discharge from care. Rula Health is known for both retention and appropriate transitions with data-driven monitoring via MBC and established referral pathways. However, distinguishing between premature dropout versus clinically appropriate transition is critical, as each has different implications for continuity and safety. Future research should use methods, such as survival analyses and qualitative follow-up, to clarify reasons for disengagement and should further investigate the mechanisms underlying engagement (eg, perceptions of safety, trust, and therapeutic alliance) to better understand how adolescents interact with digital care and inform strategies to support continuity of care.

Rula Health’s model, which included personalized intake and MBC integration, decreased depression and anxiety symptoms over the course of 12 visits across the entire adolescent sample. Both groups showed clinically meaningful improvements, with average reductions meeting or exceeding established MCID thresholds. This study is one of the few real-world evaluations of the effects of a commercial MBC-based DMHI on depression and anxiety symptom trajectories in adolescents with and without suicide risk. Recent research shows that virtual therapy is as effective as face-to-face treatment for adolescents with anxiety and depression [24,71]. However, few studies have disaggregated outcomes by suicide risk status or included high-risk populations due to safety and liability concerns [29,72]. Our findings address these gaps by demonstrating that a virtual therapy DMHI, Rula Health, decreases depression and anxiety symptoms among adolescents with varying levels of suicide risk in the real world. Future research should identify which specific components of Rula Health’s digital platform (eg, personalized matching, MBC, or their combination) most strongly contribute to treatment effectiveness in this real-world setting.

MBC-based DMHIs (such as Rula Health) have the potential to deliver rapid, meaningful clinical improvement for high-risk youth. Adolescents with elevated suicide risk experienced a decrease in both anxiety and depression symptoms at a greater rate than adolescents with no suicide risk. The elevated suicide risk group also achieved similar depression and anxiety scores as the no-risk group by 12 visits, after accounting for demographic and clinical characteristics. These findings align with previous research showing that adolescents with severe symptoms and suicidality can improve rapidly in outpatient treatment, often at rates comparable to those with lower symptom severity or no suicide risk [16,73]. MBC delivered digitally, as in Rula Health’s model, may accelerate symptom relief in adolescents with elevated suicide risk by flagging patients who are in crisis and prompting timely adjustments to treatment. Rula Health patients can select mental health care providers based on characteristics such as demographics and clinical specialty, which may enhance therapeutic alliance, an especially important factor for individuals experiencing suicidality [74]. Alternatively, the differences between risk groups might partly reflect regression to the mean, where those starting with more severe symptoms tend to show greater improvement over time regardless of treatment [75]. Although our mixed-effects models account for individual baseline severity, regression to the mean remains a plausible alternative explanation. Future research should use designs that can disentangle regression to the mean from treatment effects and more directly test the unique contribution of MBC to adolescent outcomes.

### Strengths and Limitations

This study had several strengths. First, the large, naturalistic sample of more than 6000 adolescents from Rula Health, a widely available commercial DMHI, enhanced the generalizability of our findings. Second, this study examined treatment delivered under naturalistic conditions with real-world constraints, increasing the external validity of our findings compared to research in controlled settings. Third, the inclusion of adolescents with elevated suicide risk offers rare outcomes data on this important group that is usually underrepresented in DMHI research due to safety or liability concerns [29,72]. Finally, collecting MBC measures at regular intervals and using validated clinical outcomes measures (PHQ-9 and GAD-7) enabled us to model symptom trajectories instead of less nuanced pre-post analyses.

This study also had limitations; it lacked a more thorough assessment of suicidality, including the range of suicidal ideation and behaviors, as well as nonsuicidal self-injury. This study also lacked additional outcomes that could be important for adolescents, such as functioning at school and home, stress, and substance use. This study’s naturalistic design involved collecting neither data on specific treatment content, therapy modality, or fidelity to evidence-based practices nor other potentially important variables, such as treatment completion, dropout, or referrals. This means we could not assess either the variability in how care was delivered across mental health care providers or true attrition. Future studies should collect data on fidelity to clarify how treatment delivery influences outcomes while balancing real-world applicability with methodological rigor. In addition, because our sample was predominantly White and non-Hispanic, future research is warranted among more diverse populations to increase the generalizability of our findings. Finally, follow-up was restricted to 12 visits without any postdischarge data. Thus, the long-term effectiveness of treatment on adolescent outcomes remains unknown. Future research should include both self-report and clinician assessments of suicide risk and collect outcomes over longer follow-up periods to establish the long-term effectiveness of DMHIs for adolescents across the suicide risk spectrum.

### Conclusions

Results from this retrospective analysis highlight the potential of commercial DMHIs, such as Rula Health, to effectively reduce depression and anxiety symptoms in adolescents across varying levels of suicide risk. Baseline demographic and clinical characteristics revealed important differences between risk groups and extended current research on growing high-risk populations. All adolescents experienced substantial symptom improvements, with those at elevated suicide risk showing steeper rates of symptom reduction and ultimately achieving similar symptom levels as their no-risk peers by 12 visits. These findings highlight the capacity of structured, virtual care platforms, such as Rula Health, to deliver timely and effective support to high-risk youth. Future research and implementation efforts should explore concrete strategies for optimizing digital personalization and MBC (eg, using machine learning to enhance mental health care provider matching and comparing different schedules for symptom monitoring), particularly by including more racially and ethnically diverse adolescent populations to ensure equity and generalizability. At the policy level, integrating DMHIs into school-based mental health programs and expanding insurance reimbursement models for virtual care could help translate these findings into scalable solutions within public health systems.

## Data Availability

The datasets generated or analyzed during this study are not publicly available, given Rula Health’s privacy policy for patient data, but aggregated and anonymized data are available from the corresponding author on reasonable request.

## Appendix

Multimedia Appendix 1 Model selection results, full results including covariate effects, and model assumption checks (Tables S1-S3 and Figure S1).

## Abbreviations

C-SSRS: Columbia-Suicide Severity Rating Scale
DMHI: digital mental health intervention
GAD-7: Generalized Anxiety Disorder-7
MBC: measurement-based care
MCID: minimal clinically important difference
PHQ-9: Patient Health Questionnaire-9
PSCR: patient safety and clinical risk
