# Supplementary material for: Real-World Symptom Trajectories in Adolescents With and Without Suicide Risk Receiving Care from Rula Health: Retrospective Study
Source: JMIR Pediatr Parent. 2025 Oct 15;8:e81444. doi: 10.2196/81444 (PMC12572749; doi:10.2196/81444)
Supplement: Multimedia Appendix 1 [file pediatrics_v8i1e81444_app1.docx]

Real-World Symptom Trajectories in Adolescents With and Without Suicide Risk: A Retrospective Study of Rula Health

Supplemental Methods and Results

*Model Selection Results:*

| Table S1  Selection of Optimal Mixed Model | | | | | | |
| --- | --- | --- | --- | --- | --- | --- |
| Consecutive Steps & Tested Effect | Unadjusted Models | | | Adjusted Models^a^ | | |
| Outcome: PHQ-9 Score | χ^2^ | df | p | χ^2^ | df | p |
| 1. Main Effect of Visit # | 2663.4 | 2 | <.0001 | 1443.2 | 2 | <.0001 |
| 2. Main Effect of Suicide Risk Group | 987.02 | 1 | <.0001 | 422.27 | 1 | <.0001 |
| 3. Interaction of Suicide Risk Group with Visit # | 171.49 | 1 | <.0001 | 76.27 | 1 | <.0001 |
| Outcome: GAD-7 Score | χ^2^ | df | p | χ^2^ | df | p |
| 1. Main Effect of Visit # | 2552.7 | 2 | <.0001 | 1407.3 | 2 | <.0001 |
| 2. Main Effect of Suicide Risk Group | 466.44 | 1 | <.0001 | 205.8 | 1 | <.0001 |
| 3. Interaction of Suicide Risk Group with Visit # | 72.71 | 1 | <.0001 | 36.24 | 1 | <.0001 |
| ^a^Adjusted for age, gender, race, ethnicity, primary diagnosis, and comorbidity | | | | | | |

*Full Results Including Covariate Effects:*

Greater age was associated with greater depression and anxiety scores, on average throughout the 12 visits. Male gender was associated with lower depression and anxiety scores, on average throughout the 12 visits. Adolescents with a depressive disorder diagnosis had greater depression scores than adolescents with all other diagnoses. And adolescents with an anxiety disorder had higher anxiety scores than adolescents with all other diagnoses (although these effects were smaller than those for depression). African American/Black race was associated with lower depression and anxiety scores than White race. No other racial differences were found. Having at least one baseline comorbidity was associated with higher anxiety and depression scores (Table S2).

| Table S2  Results (fixed effects) for Best Fitting Models for Depression and Anxiety Symptom Trajectories including Covariates | | | | | | |
| --- | --- | --- | --- | --- | --- | --- |
|  | Adjusted PHQ-9 Model | | | Adjusted GAD-7 Model | | |
|  | B (SE) | t | p | B (SE) | t | p |
| Intercept | 10.36 (.30) | 34.75 | <.0001 | 9.12 (.23) | 39.48 | <.0001 |
| Visit Number | -.39 (.024) | -16.37 | <.0001 | -.35 (.020) | -17.87 | <.0001 |
| Suicide Risk Group (At Risk) | 4.95 (.22) | 22.88 | <.0001 | 2.83 (.18) | 15.74 | <.0001 |
| Visit Number x Suicide Risk Group (At Risk) | -.32 (.036) | -8.76 | <.0001 | -.18 (.030) | -6.03 | <.0001 |
| Age | .40 (.08) | 5.01 | <.0001 | .28 (.066) | 4.31 | <.0001 |
| Gender (Male) | -1.87 (.21) | -8.80 | <.0001 | -1.52 (.17) | -8.71 | <.0001 |
| Primary Diagnosis (Depressive Disorder Reference for PHQ-9 model and Anxiety Disorder reference for GAD-7 model) | | | | | | |
| Anxiety Disorder (PHQ model); Depressive Disorder (GAD model) | -.82 (.20) | -4.19 | <.0001 | -.34 (.17) | -2.03 | .042 |
| Trauma and Stress Related Disorder | -1.00 (.23) | -4.32 | <.0001 | -.58 (.18) | -3.19 | .0014 |
| Other Diagnosis | -1.01 (.22) | -4.68 | <.0001 | -.70 (.18) | -3.97 | <.0001 |
| Race (White Reference) | | | | | | |
| African American/Black | -1.09 (.35) | -3.14 | .017 | -.82 (.28) | -2.89 | .0039 |
| American-Indian or Alaska Native | 2.02 (.95) | 2.12 | .034 | .27 (.78) | .34 | .73 |
| Asian | -.15 (.37) | -.40 | .69 | -.33 (.31 | -1.07 | .29 |
| Mixed Race | .037 (.27) | .14 | .89 | -.074 (.22) | -.34 | .74 |
| Native Hawaiian or Pacific Islander | .033 (.67) | .049 | .96 | -.13 (.56) | -.23 | .82 |
| Ethnicity (Not Hispanic) | .28 (.24) | 1.17 | .24 | .19 (.20) | .96 | .34 |
| Baseline Comorbidity (Present) | 1.03 (.15) | 6.86 | <.0001 | .89 (.13) | 6.99 | <.0001 |

*Model Assumption Checks:*

Generalized variance inflation factors (GVIFs) were calculated to assess multicollinearity, with all values below 5.0, indicating acceptable levels of multicollinearity (Table S3). Normality of residuals was assessed through Shapiro-Wilk tests and visual inspection of residual Q-Q plots and histograms. Results from the Shapiro-Wilk tests indicated deviations from normality for the PHQ-9 model (W=.99, p<.0001) and the GAD-7 model (W=.99, p < .001), however Q-Q plots and histograms showed approximately normal residual distributions with minor deviations only in the extreme tails (Figure S1). Given the robustness of multilevel models to minor violations of assumptions [[1]](https://www.zotero.org/google-docs/?F9CktF) and our large sample size, the final models were retained.

| Table S3  Variable Inflation Factors for Model Predictors | | |
| --- | --- | --- |
| Variable | PHQ-9 Model | GAD-7 Model |
| Visit Number | 1.76 | 1.76 |
| Suicide Risk Group | 1.18 | 1.21 |
| Visit Number x Suicide Risk Group | 1.91 | 1.93 |
| Age | 1.02 | 1.02 |
| Gender | 1.02 | 1.02 |
| Primary Diagnosis | 1.09 | 1.09 |
| Race | 1.34 | 1.34 |
| Ethnicity | 1.31 | 1.31 |
| Baseline Comorbidity | 1.05 | 1.06 |

| Figure S1  Q-Q Plots and Hisograms of Model Residuals | |
| --- | --- |
| 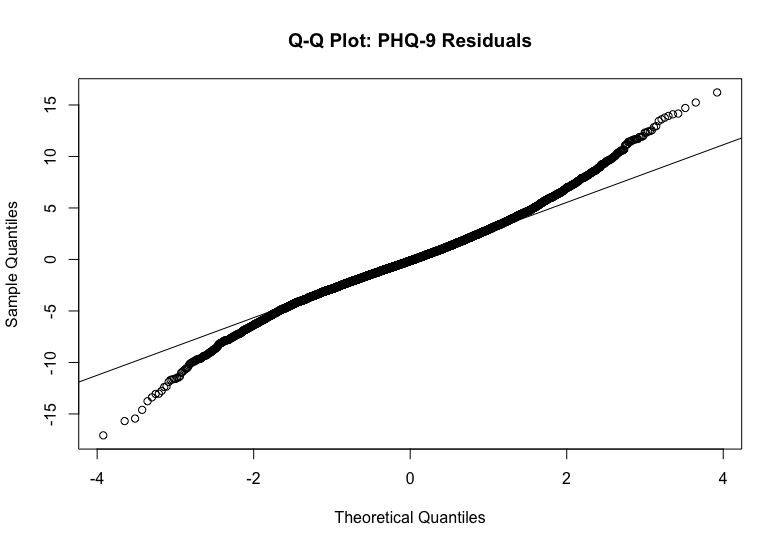 | 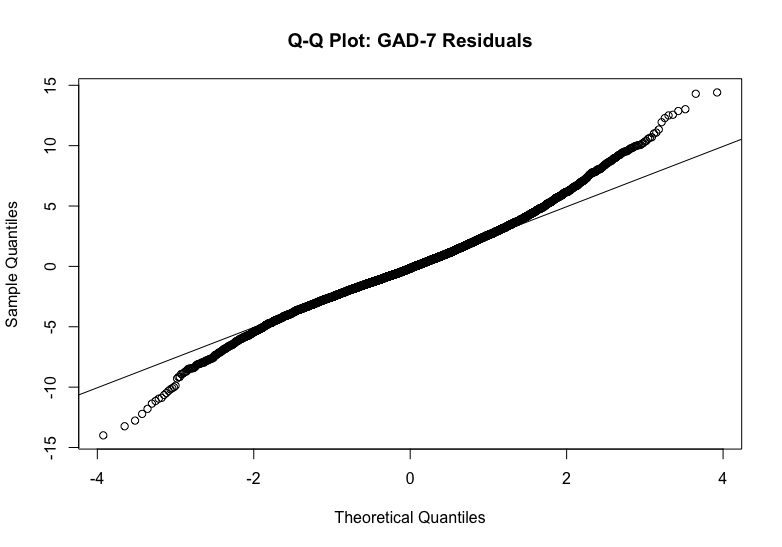 |
| 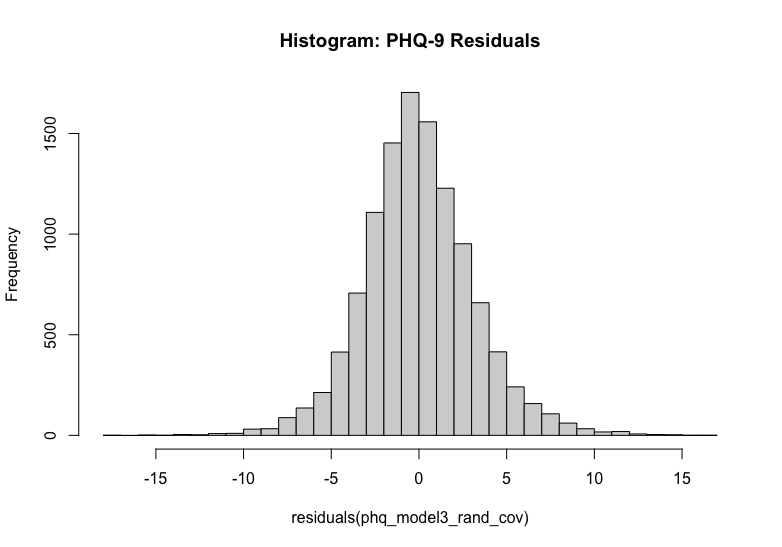 | 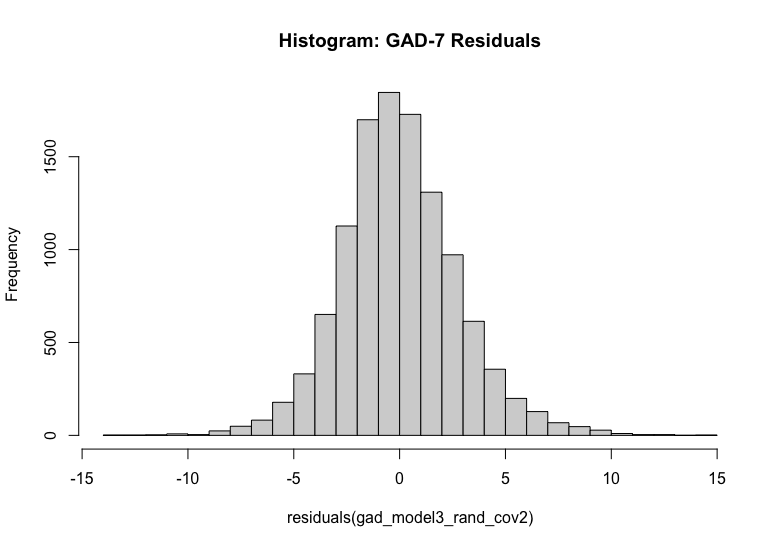 |

References:

[1.](https://www.zotero.org/google-docs/?t8H67W)  [Schielzeth H, Dingemanse NJ, Nakagawa S, Westneat DF, Allegue H, Teplitsky C, Réale D, Dochtermann NA, Garamszegi LZ, Araya-Ajoy YG. Robustness of linear mixed-effects models to violations of distributional assumptions. Methods Ecol Evol 2020;11(9):1141–1152. doi: 10.1111/2041-210X.13434](https://www.zotero.org/google-docs/?t8H67W)
